# *N*-(5-Phenyl-1*H*-pyrazol-3-yl)benzene-1,2-diamine

**DOI:** 10.1107/S1600536810009104

**Published:** 2010-03-17

**Authors:** Mohamadou Lamine Doumbia, Rachid Bouhfid, El Mokhtar Essassi, Lahcen El Ammari

**Affiliations:** a Laboratoire de Chimie Organique Hétérocyclique, Pôle de Compétences, Pharmacochimie, Av Ibn Battouta, BP 1014, Faculté des Sciences, Université Mohammed V-Agdal, Rabat, Morocco; b Institute of Nanomaterials and Nanotechnology, Avenue Armée Royale, Rabat, Morocco; c Laboratoire de Chimie du Solide Appliquée, Faculté des Sciences, Université Mohammed V-Agdal, Avenue Ibn Battouta, BP 1014, Rabat, Morocco

## Abstract

In the title compound, C_15_H_14_N_4_, the phenyl and pyrazole rings are essentially coplanar, being twisted relative to each other by a dihedral of only 3.68 (11)°. The benzene ring makes a dihedral angle of 64.47 (11)° with the pyrazole ring. The crystal structure is stabilized by two inter­molecular N—H⋯N hydrogen-bonds, which build a two-dimensional network developing parallel to (100). An intra­molecular N—H⋯N hydrogen bond also occurs.

## Related literature

For the pharmacological applications of *N*-(3-phenyl-1*H*-pyrazol-5-yl)benzene-1, 2-diamine, see: Sharon *et al.* (2005 ▶); Barsoum *et al.* (2006 ▶); Cunico *et al.* (2006 ▶). For the use of pyrazole derivatives as chelating agents, see: Onishi *et al.* (2006 ▶) and as corrosion inibitors, see: Tebbji *et al.* (2005 ▶).
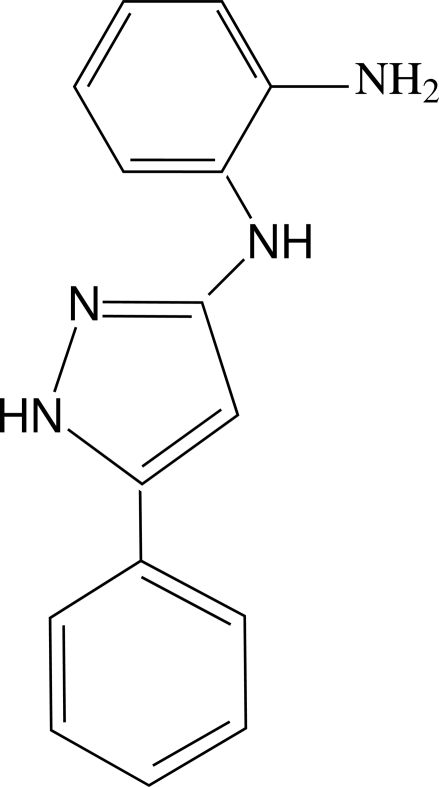


## Experimental

### 

#### Crystal data

C_15_H_14_N_4_
*M* *_r_* = 250.30
Monoclinic, 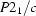

*a* = 13.2357 (8) Å
*b* = 5.8473 (4) Å
*c* = 16.4039 (10) Å
β = 92.074 (4)°
*V* = 1268.72 (14) Å^3^
*Z* = 4
Mo *K*α radiation
μ = 0.08 mm^−1^
*T* = 298 K
0.32 × 0.27 × 0.19 mm

#### Data collection

Bruker X8 APEXII CCD area-detector diffractometer
11640 measured reflections
2333 independent reflections
1527 reflections with *I* > 2σ(*I*)
*R*_int_ = 0.056

#### Refinement

*R*[*F* ^2^ > 2σ(*F* ^2^)] = 0.046
*wR*(*F* ^2^) = 0.127
*S* = 1.02
2333 reflections
228 parameters
H atoms treated by a mixture of independent and constrained refinement
Δρ_max_ = 0.15 e Å^−3^
Δρ_min_ = −0.15 e Å^−3^

### 

Data collection: *APEX2* (Bruker, 2005 ▶); cell refinement: *SAINT* (Bruker, 2005 ▶); data reduction: *SAINT* (Bruker, 2005 ▶); program(s) used to solve structure: *SHELXS97* (Sheldrick, 2008 ▶); program(s) used to refine structure: *SHELXL97* (Sheldrick, 2008 ▶); molecular graphics: *ORTEP-3 for Windows* (Farrugia, 1997 ▶) and *PLATON* (Spek, 2009 ▶); software used to prepare material for publication: *WinGX* (Farrugia, 1999 ▶).

## Supplementary Material

Crystal structure: contains datablocks global, I. DOI: 10.1107/S1600536810009104/dn2548sup1.cif

Structure factors: contains datablocks I. DOI: 10.1107/S1600536810009104/dn2548Isup2.hkl

Additional supplementary materials:  crystallographic information; 3D view; checkCIF report

## Figures and Tables

**Table 1:** Hydrogen-bond geometry (Å, °)

*D*—H⋯*A*	*D*—H	H⋯*A*	*D*⋯*A*	*D*—H⋯*A*
N1—H1⋯N2^i^	0.97 (2)	2.14 (3)	3.048 (2)	157 (2)
N3—H3⋯N4^ii^	0.89 (2)	2.27 (2)	3.122 (2)	160 (2)
N4—H4*B*⋯N2	0.95 (3)	2.36 (3)	3.086 (2)	132 (2)

Symmetry codes: (i) 
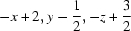
; (ii) 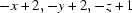.

**Fig. 1.**
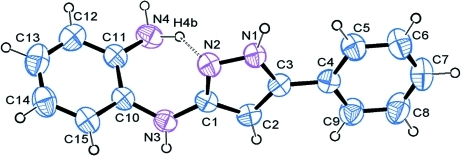
: The title molecule with the atom-labeling scheme. The displacement ellipsoids are drawn at the 50% probability level. H atoms are represented as small sphere of arbitray radii.

**Fig. 2.**
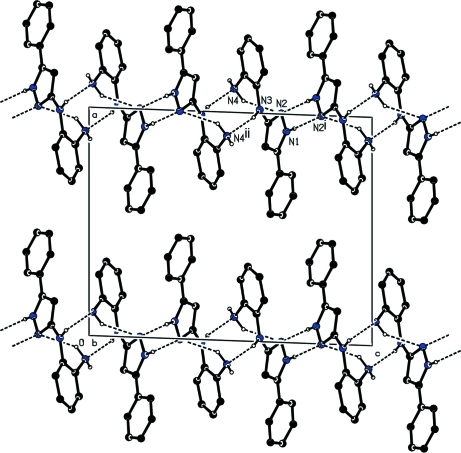
: Packing view showing the N—H···N interactions as dashed lines. H atoms not involved in hydrogen bonding have been omitted for clarity. [Symmetry codes: (i) -x+2, y-1/2, -z+3/2; (ii) -x+2, -y+2, -z+1]

## supplementary crystallographic information

### Comment

Pyrazole derivatives have attracted particular interest during the last years due to the use of such ring systems as the core structure of many drug substances, covering wide range of pharmacological applications. They were reported to possess antihyperglycemic (Sharon *et al.*, 2005), anti-inflammatory (Barsoum *et al.*, 2006) and antimalarial activitys (Cunico *et al.*, 2006). Further, pyrazole derivatives is also, used as chelating agent (Onishi *et al.*, 2006) and inhibitor of the corrosion of the steel (Tebbji *et al.*, 2005).

The *N*-(3-phenyl-1*H*-pyrazol-5-yl)benzene-1,2-diamine molecule structure is built up from three rings (phenyl, pyrazol and benzene) interconnected like linear chain as schown in Fig. 1. The phenyl and pyrazol rings are essentially planar and are only twisted to each other by a dihedral of 3.68 (11)°. As schown in Fig. 1, the molecule is not planar and the dihedral angle between the phenyl and pyrazol rings mean plane and the benzene ring is 64.21 (9)°. Two intermolecular N—H···N hydrogen bonds ensures the cohesion of the crystal structure building up a two dimensional network parallel to the (1 0 0) plane (Table 1, Fig.2).

### Experimental

A solution of (1 g, 3,96 mmol) 4-phenyl-1,5-benzodiazepine-2-thione and (1.1 ml, 15.85 mmol) of hydrate bhydrazine in 20 ml of ethanol was refluxed for 4 h. The solvent was removed in vaccuo and the residue was washed with 60 ml of water. The resulting product was recrystallized from ethanol to give *N*-(3-phenyl-1*H*-pyrazol-5-yl)benzene-1,2-diamine in 60% yield..

### Refinement

All H atoms were located in a difference map and refined.

### Crystal data

**Table:**

C_15_H_14_N_4_	*F*(000) = 528
*M**_r_* = 250.30	*D*_x_ = 1.310 Mg m^−^^3^
Monoclinic, *P*2_1_/*c*	Mo *K*α radiation, λ = 0.71073 Å
Hall symbol: -p 2ybc	Cell parameters from 12462 reflections
*a* = 13.2357 (8) Å	θ = 25.4–2.5°
*b* = 5.8473 (4) Å	µ = 0.08 mm^−^^1^
*c* = 16.4039 (10) Å	*T* = 298 K
β = 92.074 (4)°	Parallelepiped, clear pale yellow
*V* = 1268.72 (14) Å^3^	0.32 × 0.27 × 0.19 mm
*Z* = 4	

### Data collection

**Table:**

Bruker X8 APEX CCD area-detector diffractometer	1527 reflections with *I* > 2σ(*I*)
Radiation source: fine-focus sealed tube	*R*_int_ = 0.056
graphite	θ_max_ = 25.4°, θ_min_ = 2.5°
φ and ω scans	*h* = −15→15
11640 measured reflections	*k* = −7→6
2333 independent reflections	*l* = −19→19

### Refinement

**Table:**

Refinement on *F*^2^	Primary atom site location: structure-invariant direct methods
Least-squares matrix: full	Secondary atom site location: difference Fourier map
*R*[*F*^2^ > 2σ(*F*^2^)] = 0.046	Hydrogen site location: inferred from neighbouring sites
*wR*(*F*^2^) = 0.127	H atoms treated by a mixture of independent and constrained refinement
*S* = 1.02	*w* = 1/[σ^2^(*F*_o_^2^) + (0.0719*P*)^2^] where *P* = (*F*_o_^2^ + 2*F*_c_^2^)/3
2333 reflections	(Δ/σ)_max_ < 0.001
228 parameters	Δρ_max_ = 0.15 e Å^−^^3^
0 restraints	Δρ_min_ = −0.15 e Å^−^^3^

### Special details

**Table:**

Geometry. All esds (except the esd in the dihedral angle between two l.s. planes) are estimated using the full covariance matrix. The cell esds are taken into account individually in the estimation of esds in distances, angles and torsion angles; correlations between esds in cell parameters are only used when they are defined by crystal symmetry. An approximate (isotropic) treatment of cell esds is used for estimating esds involving l.s. planes.
Refinement. Refinement of *F*^2^ against ALL reflections. The weighted *R*-factor wR and goodness of fit *S* are based on *F*^2^, conventional *R*-factors *R* are based on *F*, with *F* set to zero for negative *F*^2^. The threshold expression of *F*^2^ > σ(*F*^2^) is used only for calculating *R*-factors(gt) etc. and is not relevant to the choice of reflections for refinement. *R*-factors based on *F*^2^ are statistically about twice as large as those based on *F*, and *R*- factors based on ALL data will be even larger.

### Fractional atomic coordinates and isotropic or equivalent isotropic displacement parameters (Å^2^)

**Table:**

	*x*	*y*	*z*	*U*_iso_*/*U*_eq_	
N1	0.92397 (12)	0.5307 (3)	0.69736 (9)	0.0414 (4)	
N2	1.00286 (12)	0.6719 (3)	0.67931 (9)	0.0423 (4)	
N3	1.01564 (13)	1.0130 (3)	0.60311 (9)	0.0426 (4)	
N4	1.10392 (16)	0.6520 (3)	0.51288 (11)	0.0507 (5)	
C1	0.95922 (14)	0.8361 (3)	0.63347 (10)	0.0369 (5)	
C2	0.85506 (15)	0.8033 (4)	0.62309 (11)	0.0416 (5)	
C3	0.83439 (14)	0.6054 (3)	0.66539 (10)	0.0385 (5)	
C4	0.73944 (15)	0.4805 (4)	0.67390 (11)	0.0421 (5)	
C5	0.73429 (19)	0.2767 (4)	0.71726 (13)	0.0541 (6)	
C6	0.6440 (2)	0.1608 (5)	0.72155 (16)	0.0669 (7)	
C7	0.55732 (19)	0.2441 (5)	0.68386 (16)	0.0684 (7)	
C8	0.56061 (19)	0.4460 (5)	0.64097 (17)	0.0695 (7)	
C9	0.65081 (17)	0.5631 (5)	0.63635 (14)	0.0576 (6)	
C10	1.12135 (15)	1.0028 (3)	0.59179 (10)	0.0396 (5)	
C11	1.16551 (15)	0.8230 (3)	0.54840 (11)	0.0424 (5)	
C12	1.26837 (18)	0.8308 (5)	0.53560 (13)	0.0561 (6)	
C13	1.32666 (19)	1.0142 (5)	0.56121 (15)	0.0662 (7)	
C14	1.2833 (2)	1.1923 (5)	0.60222 (14)	0.0619 (7)	
C15	1.18167 (18)	1.1845 (4)	0.61841 (12)	0.0498 (6)	
H15	1.1477 (16)	1.308 (4)	0.6454 (13)	0.056 (6)*	
H1	0.9384 (19)	0.389 (4)	0.7256 (16)	0.083 (8)*	
H2	0.8072 (16)	0.894 (4)	0.5930 (13)	0.054 (6)*	
H3	0.9810 (18)	1.130 (4)	0.5814 (15)	0.070 (8)*	
H4A	1.1389 (18)	0.521 (5)	0.4963 (15)	0.073 (8)*	
H4B	1.052 (2)	0.591 (5)	0.5453 (18)	0.092 (9)*	
H5	0.7973 (19)	0.217 (4)	0.7414 (15)	0.075 (7)*	
H6	0.6439 (18)	0.011 (5)	0.7494 (16)	0.082 (8)*	
H7	0.4918 (19)	0.165 (4)	0.6883 (15)	0.074 (7)*	
H8	0.498 (2)	0.513 (4)	0.6133 (16)	0.089 (8)*	
H9	0.6532 (19)	0.707 (5)	0.6077 (16)	0.082 (8)*	
H12	1.2969 (19)	0.700 (4)	0.5093 (15)	0.074 (8)*	
H13	1.4002 (19)	1.025 (4)	0.5512 (15)	0.074 (7)*	
H14	1.3233 (18)	1.322 (4)	0.6203 (14)	0.067 (7)*	

### Atomic displacement parameters (Å^2^)

**Table:**

	*U*^11^	*U*^22^	*U*^33^	*U*^12^	*U*^13^	*U*^23^
N1	0.0454 (10)	0.0454 (11)	0.0333 (8)	−0.0028 (8)	−0.0006 (7)	0.0066 (8)
N2	0.0474 (10)	0.0455 (11)	0.0338 (8)	−0.0027 (8)	−0.0025 (7)	0.0044 (7)
N3	0.0481 (11)	0.0403 (11)	0.0392 (9)	0.0005 (9)	0.0011 (7)	0.0042 (8)
N4	0.0659 (13)	0.0445 (12)	0.0418 (10)	−0.0001 (11)	0.0015 (9)	−0.0055 (8)
C1	0.0472 (12)	0.0379 (12)	0.0255 (9)	−0.0001 (9)	0.0006 (7)	−0.0004 (8)
C2	0.0474 (13)	0.0451 (13)	0.0320 (9)	0.0064 (10)	−0.0016 (8)	0.0016 (9)
C3	0.0431 (11)	0.0461 (13)	0.0261 (9)	0.0016 (9)	−0.0008 (8)	−0.0016 (8)
C4	0.0469 (12)	0.0469 (14)	0.0326 (9)	−0.0004 (10)	0.0021 (8)	−0.0056 (9)
C5	0.0567 (15)	0.0555 (16)	0.0498 (12)	−0.0048 (12)	−0.0028 (10)	0.0050 (11)
C6	0.0655 (17)	0.0678 (19)	0.0673 (16)	−0.0164 (14)	0.0007 (13)	0.0101 (14)
C7	0.0525 (16)	0.082 (2)	0.0707 (16)	−0.0185 (15)	0.0025 (12)	0.0020 (14)
C8	0.0454 (15)	0.088 (2)	0.0750 (17)	−0.0034 (14)	−0.0012 (12)	0.0110 (15)
C9	0.0491 (14)	0.0641 (17)	0.0593 (14)	0.0001 (12)	−0.0010 (10)	0.0074 (13)
C10	0.0480 (12)	0.0425 (13)	0.0280 (9)	−0.0022 (10)	−0.0028 (8)	0.0044 (8)
C11	0.0518 (13)	0.0447 (13)	0.0305 (9)	−0.0017 (10)	−0.0018 (8)	0.0053 (9)
C12	0.0562 (15)	0.0675 (17)	0.0447 (12)	0.0069 (14)	0.0018 (10)	0.0055 (12)
C13	0.0498 (15)	0.090 (2)	0.0590 (15)	−0.0070 (15)	−0.0007 (12)	0.0108 (14)
C14	0.0614 (16)	0.0685 (19)	0.0551 (14)	−0.0175 (15)	−0.0079 (12)	0.0046 (13)
C15	0.0586 (15)	0.0505 (15)	0.0400 (11)	−0.0076 (12)	−0.0035 (10)	0.0028 (10)

### Geometric parameters (Å, °)

**Table:**

N1—C3	1.352 (2)	C6—C7	1.372 (4)
N1—N2	1.372 (2)	C6—H6	0.99 (3)
N1—H1	0.96 (3)	C7—C8	1.376 (4)
N2—C1	1.338 (2)	C7—H7	0.99 (2)
N3—C1	1.379 (2)	C8—C9	1.381 (3)
N3—C10	1.419 (2)	C8—H8	1.01 (3)
N3—H3	0.89 (2)	C9—H9	0.96 (3)
N4—C11	1.404 (3)	C10—C15	1.390 (3)
N4—H4A	0.94 (3)	C10—C11	1.408 (3)
N4—H4B	0.96 (3)	C11—C12	1.386 (3)
C1—C2	1.396 (3)	C12—C13	1.378 (4)
C2—C3	1.382 (3)	C12—H12	0.96 (2)
C2—H2	0.95 (2)	C13—C14	1.376 (4)
C3—C4	1.465 (3)	C13—H13	1.00 (2)
C4—C5	1.391 (3)	C14—C15	1.382 (3)
C4—C9	1.392 (3)	C14—H14	0.97 (3)
C5—C6	1.378 (3)	C15—H15	0.96 (2)
C5—H5	0.97 (3)		
			
C3—N1—N2	112.68 (17)	C6—C7—C8	119.6 (3)
C3—N1—H1	128.2 (16)	C6—C7—H7	121.5 (14)
N2—N1—H1	118.8 (15)	C8—C7—H7	118.9 (14)
C1—N2—N1	103.70 (15)	C7—C8—C9	119.8 (2)
C1—N3—C10	124.40 (17)	C7—C8—H8	121.6 (15)
C1—N3—H3	116.4 (15)	C9—C8—H8	118.6 (15)
C10—N3—H3	118.3 (15)	C8—C9—C4	121.3 (2)
C11—N4—H4A	114.5 (15)	C8—C9—H9	120.2 (15)
C11—N4—H4B	117.1 (16)	C4—C9—H9	118.4 (15)
H4A—N4—H4B	103 (2)	C15—C10—C11	119.0 (2)
N2—C1—N3	120.85 (17)	C15—C10—N3	118.98 (19)
N2—C1—C2	111.97 (17)	C11—C10—N3	121.78 (17)
N3—C1—C2	127.18 (18)	C12—C11—N4	121.3 (2)
C3—C2—C1	105.46 (17)	C12—C11—C10	118.7 (2)
C3—C2—H2	125.9 (13)	N4—C11—C10	119.76 (19)
C1—C2—H2	128.6 (13)	C13—C12—C11	121.5 (2)
N1—C3—C2	106.18 (18)	C13—C12—H12	122.1 (15)
N1—C3—C4	123.06 (18)	C11—C12—H12	116.4 (15)
C2—C3—C4	130.67 (18)	C14—C13—C12	120.0 (2)
C5—C4—C9	117.9 (2)	C14—C13—H13	117.3 (14)
C5—C4—C3	122.29 (19)	C12—C13—H13	122.7 (14)
C9—C4—C3	119.8 (2)	C13—C14—C15	119.7 (3)
C6—C5—C4	120.4 (2)	C13—C14—H14	120.9 (14)
C6—C5—H5	122.4 (15)	C15—C14—H14	119.5 (14)
C4—C5—H5	117.2 (15)	C14—C15—C10	121.1 (2)
C7—C6—C5	121.0 (3)	C14—C15—H15	122.5 (13)
C7—C6—H6	120.6 (15)	C10—C15—H15	116.3 (13)
C5—C6—H6	118.3 (15)		

### Hydrogen-bond geometry (Å, °)

**Table:**

*D*—H···*A*	*D*—H	H···*A*	*D*···*A*	*D*—H···*A*
N1—H1···N2^i^	0.97 (2)	2.14 (3)	3.048 (2)	157 (2)
N3—H3···N4^ii^	0.89 (2)	2.27 (2)	3.122 (2)	160 (2)
N4—H4B···N2	0.95 (3)	2.36 (3)	3.086 (2)	132 (2)

Symmetry codes: (i) −*x*+2, *y*−1/2, −*z*+3/2; (ii) −*x*+2, −*y*+2, −*z*+1.
